# A case of biliary atresia with pancreaticobiliary maljunction

**DOI:** 10.1186/s40792-017-0375-2

**Published:** 2017-09-05

**Authors:** Kosuke Endo, Akiko Yokoi, Yasuhiko Mishima, Akihiko Tamaki, Keiichi Morita, Yuichi Okata, Chieko Hisamatsu, Hiroaki Fukuzawa, Makiko Yoshida, Yoshinobu Akasaka, Kosaku Maeda

**Affiliations:** 1 Department of Pediatric Surgery, Kobe Children’s Hospital, 1-6-7 Minatojima Minamimachi, Chuo-ku, Kobe, Hyogo 654-0081 Japan; 2 Department of Pediatric Surgery, Kitano Hospital The Tazuke Kofukai Medical Research Institute, 2-4-20 Ohgimachi, Kita-Ku, Osaka, 530-8480 Japan; 3 Department of Radiology, Kobe Children’s Hospital, 1-6-7 Minatojima Minamimachi, Chuo-ku, Kobe, Hyogo 654-0081 Japan; 4 Department of Pathology, Kobe Children’s Hospital, 1-6-7 Minatojima Minamimachi, Chuo-ku, Kobe, Hyogo 654-0081 Japan

**Keywords:** Biliary atresia, Ultrasonography, Gallbladder contraction, Patent common bile duct type, Pancreaticobiliary maljunction

## Abstract

**Background:**

The pathogenesis of biliary atresia (BA) is still unknown. There are several reports on the etiology of BA, including pancreaticobiliary maljunction (PBM). We experienced a case of Kasai type IIIa BA with PBM, in which we found elevation of pancreatic enzymes in the gallbladder. We evaluated whether PBM is related to the pathogenesis of BA based on our findings.

**Case presentation:**

The patient was born at 40 weeks of gestation. His body weight at birth was 2850 g. At the age of 4 days, he had an acholic stool and was referred to our hospital. Abdominal ultrasonography showed that triangular cord sign was negative. The gallbladder was isolated with a diameter of 19 mm, and it contracted in response to oral feeding. His ultrasonographic findings were atypical for BA, but his jaundice did not improve. Therefore, we performed an operation at the age of 56 days. Intraoperative cholangiography showed a common bile duct and pancreatic duct and a common channel patent, while the common hepatic duct or intrahepatic duct was not visualized. Bile in the gallbladder contained colorless fluid, which showed elevated lipase level (34,100 IU/L). We performed Kasai portoenterostomy under the diagnosis of Kasai type IIIa BA with PBM. The patient’s postoperative course was uneventful, and he was discharged on day 30 after the operation. Histopathological evaluation showed that the lumens of the common bile duct and cystic duct were patent. However, the common hepatic duct was closed, and only bile ductules with diameters of less than 50 μm were isolated. Infiltration of lymphocytes was detected in the porta hepatis. No apparent inflammation was observed around the cystic duct, which was constantly exposed to pancreatic juice because of reflux through PBM.

**Conclusions:**

Reflux of pancreatic juice through PBM might not be an etiological factor for BA, but might be associated with patency of the common and cystic bile ducts in Kasai type IIIa BA.

## Background

The pathogenesis of biliary atresia (BA) remains unknown. There have been several reports on the etiology of BA [1–7]. Landing [8] proposed the concept of “infantile obstructive cholangiopathy” as a common cause of the pathogenesis of neonatal hepatitis, choledochal cyst, and BA. This concept of obstructive cholangiopathy was based on the findings of pancreaticobiliary maljunction (PBM). PBM is found in most cases of congenital dilatation of the bile duct [9]. However, there have only been a few reports [10, 11] on the arrangement of the pancreaticobiliary duct in cases of BA. We experienced a case of BA with PBM in which we found elevation of pancreatic enzymes in the gallbladder (GB). We evaluated how PBM relates to the pathogenesis of BA based on our findings.

## Case presentation

The patient was born at 40 weeks of gestation. His body weight at birth was 2850 g. At the age of 4 days, an acholic stool was observed and he was brought to a nearby clinic. His acholic stool did not improve, and laboratory data showed elevation of bilirubin levels. Therefore, he was referred to our hospital for further evaluation.

**Fig. 1:**
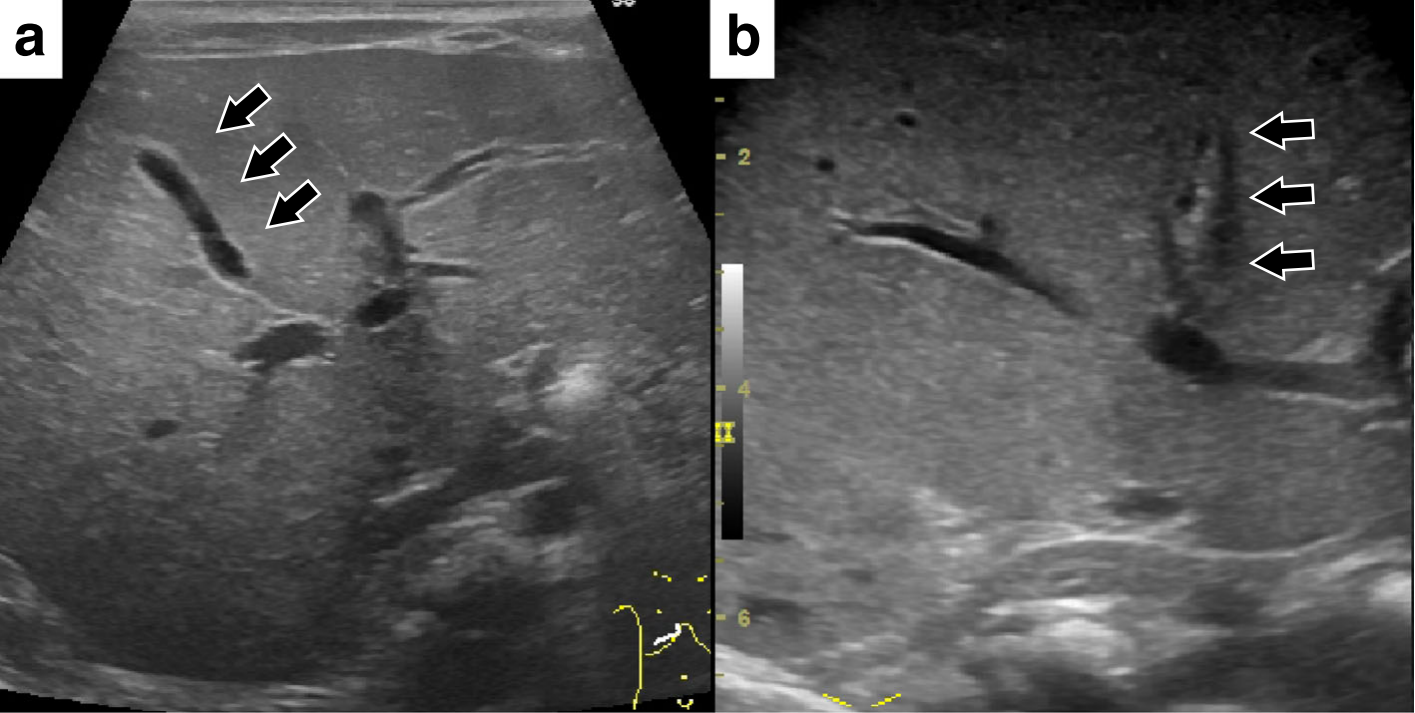
Preoperative abdominal ultrasonography. **a** Six hours after oral feeding. A gallbladder with a diameter of 19 mm was isolated. **b** The gallbladder contracted 1.5 h after oral feeding

His general condition was good except for his jaundice. Laboratory data showed an elevation in the direct bilirubin level (T-Bil/D-Bil, 6.05/3.72 mg/dL). Immunoglobulin M antibodies for Toxoplasma, Rubella, Cytomegalovirus, Herpes simplex, and Syphilis were all negative. Abdominal ultrasonography (US) showed negative triangular cord signs, and the GB was 19 mm in size and contracted in response to oral feeding (Fig. 1). We repeated abdominal US several times, but the results were the same. However, his jaundice did not improve. Therefore, we performed open biopsy and cholangiography at the age of 56 days.

**Fig. 2:**
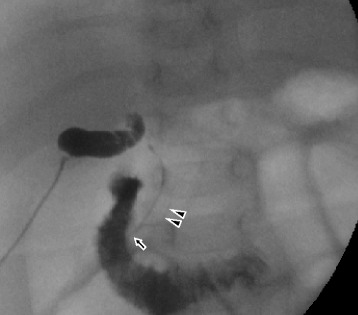
Intraoperative cholangiogram. A common channel (arrow), common bile duct, and pancreatic duct (arrowhead) can be seen

The GB contained colorless fluid, and amylase and lipase levels of it were 203 and 34,100 IU/L, respectively. A thin mass of connective tissue was observed in the porta hepatis. Cholangiography showed a common bile duct and pancreatic duct and a common channel patent, but a common hepatic duct or intrahepatic duct was not observed (Fig. 2). We performed Kasai portoenterostomy under the diagnosis of Kasai type IIIa BA with PBM [12]. The postoperative course was uneventful, and he was discharged on the 30th day after the operation. Two years after the operation, he is free of jaundice with his native liver.

**Fig. 3:**
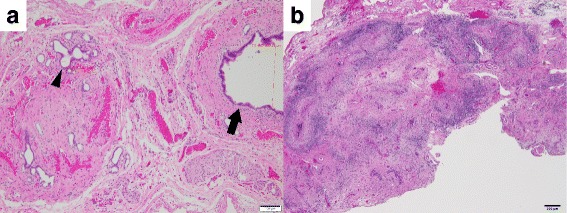
Histopathological findings (hematoxylin–eosin staining). **a** Common hepatic duct and cystic duct. The diameter of the cystic duct was 300 μm (arrow), while the common hepatic duct was closed and only bile ductules with a diameter of less than 50 μm were isolated (arrowhead). No apparent inflammation was observed around the cystic duct. The scale bar represents 100 μm. **b** Connective tissue in the porta hepatis. Severe infiltration of lymphocytes was detected. The scale bar represents 200 μm

Histopathological evaluation showed that the lumens of the common bile duct and cystic duct could be isolated macroscopically, and the diameter of the cystic duct was 300 μm. However, the common hepatic duct was closed, and only bile ductules with diameters of less than 50 μm were isolated (Fig. 3a). No apparent inflammation was present around the cystic duct. Severe infiltration of lymphocytes was detected in the porta hepatis (Fig. 3b).

## Conclusions

The pathogenesis of BA is still unknown. Several theories on the possible etiology of BA have been suggested, such as ischemia [2], immune-mediated mechanisms [3], and viral infection [5–7]. However, no theory can yet successfully explain the cause of obstruction of the lumen in the biliary tract. Miyano et al. reported a high incidence of PBM in patients with BA using autopsied specimens [4]. They suggested that reflux of pancreatic juice to the bile duct through PBM might cause inflammation, leading to obstruction of the biliary tract.

In our case, intraoperative cholangiography showed a pancreatic duct and common channel. In addition, bile in the GB showed elevated lipase levels, which indicated reflux of pancreatic juice into the GB. Discrepancy of the levels of pancreatic enzymes, or amylase and lipase, in the bile within the GB could be attributed to the immaturity of the pancreas of infants. Todani et al. reported that no abnormally high level of amylase in the bile within the cyst was observed in babies under the age of 12 months, while other pancreatic enzymes in the bile, such as trypsin, elastase I, and lipase, showed abnormally high levels in infants with choledochal cyst, regardless of age [13]. Deguchi et al. also reported a high incidence of PBM in subtype a BA [14]. However, to the best of our knowledge, this is the first report of elevation of pancreatic enzymes in bile of the GB in BA. The common bile duct and GB, which are constantly exposed to pancreatic juice, remained open in our patient, while the intrahepatic bile duct and hepatic duct became constricted and closed. Histopathological evaluation showed no apparent inflammation around the GB, while severe infiltration of lymphocytes at the porta hepatis was observed. The situation of the common bile duct and cystic duct being highly exposed to the pancreatic juice and remaining open, while the common hepatic duct, which is less exposed, being closed, would be contradictory. Our results suggest that reflux of the pancreatic juice due to PBM might not be an etiology of BA.

Preoperative US showed that the GB was constricted in response to oral feeding. Ikeda et al. reported that the GB sometimes constricts in response to oral feeding in subtype a BA, which could be a pitfall in the diagnosis of BA [15]. We retrospectively evaluated patients with BA who were treated in the Department of Pediatric Surgery, Kobe Children’s Hospital. Between 2006 and 2015, 35 patients were diagnosed with BA and treated. Among them, the common bile duct and duodenum were visualized by an intraoperative cholangiogram (Kasai type IIIa BA) in five cases. In three of these cases, the pancreatic duct was also visualized by cholangiogram, leading to the diagnosis of BA with PBM. Constant reflux of the pancreatic juice through PBM might contribute to the lumen of the common bile duct and cystic duct remaining open. However, further evaluation of this issue may be required.

Findings from our case indicate that reflux of pancreatic juice through PBM in BA might not be an etiological factor for atresia of the extrahepatic bile duct. PBM might be associated with patency of the common bile duct in Kasai type IIIa BA. However, further evaluation of this situation is required.

## Abbreviations

BA: Biliary atresia
Bil: Bilirubin
GB: Gallbladder
PBM: Pancreaticobiliary maljunction
US: Ultrasonography
